# Test-Taking Motivation in Education Students: Task Battery Order Affected Within-Test-Taker Effort and Importance

**DOI:** 10.3389/fpsyg.2020.559683

**Published:** 2020-11-25

**Authors:** Anett Wolgast, Nico Schmidt, Jochen Ranger

**Affiliations:** ^1^Department of Psychology, University of Applied Sciences Hannover, Hanover, Germany; ^2^Institute of Psychology, Martin-Luther-University Halle-Wittenberg, Halle, Germany

**Keywords:** expectation-value theory, diffusion modeling, latent growth curve modeling, perspective-taking, exam test-taking motivation

## Abstract

Different types of tasks exist, including tasks for research purposes or exams assessing knowledge. According to expectation-value theory, tests are related to different levels of effort and importance within a test taker. Test-taking effort and importance in students decreased over the course of high-stakes tests or low-stakes-tests in research on test-taking motivation. However, whether test-order changes affect effort, importance, and response processes of education students have seldomly been experimentally examined. We aimed to examine changes in effort and importance resulting from variations in test battery order and their relations to response processes. We employed an experimental design assessing *N* = 320 education students’ test-taking effort and importance three times as well as their performance on cognitive ability tasks and a mock exam. Further relevant covariates were assessed once such as expectancies, test anxiety, and concentration. We randomly varied the order of the cognitive ability test and mock exam. The assumption of intraindividual changes in education students’ effort and importance over the course of test taking was tested by one latent growth curve that separated data for each condition. In contrast to previous studies, responses and test response times were included in diffusion models for examining education students’ response processes within the test-taking context. The results indicated intraindividual changes in education students’ effort or importance depending on test order but similar mock-exam response processes. In particular effort did not decrease, when the cognitive ability test came first and the mock exam subsequently but significantly decreased, when the mock exam came first and the cognitive ability test subsequently. Diffusion modeling suggested differences in response processes (separation boundaries and estimated latent trait) on cognitive ability tasks suggesting higher motivational levels when the cognitive ability test came first than vice versa. The response processes on the mock exam tasks did not relate to condition.

## Introduction

Researchers analyzing data from the Programme for International Student Assessment (PISA) concerning the relations between motivation and test-taking achievement in mathematics reported that motivation explained 1–29% of the variance in achievement-test results (Kriegbaum et al., 2014). Further findings suggested item position effects on test performance (Weirich et al., 2016; Nagy et al., 2018, 2019; Rose et al., 2019; Liu and Hau, 2020). A problem found was decreased test performance over the course of taking a computer-assisted achievement test (List et al., 2017). That raised the question if motivation similarly decreased over the course of taking a computer-assisted achievement test. Researchers found low test-taking effort related to low test performance, discussed and tested several strategies for test takers’ high effort levels, for example, incentives, integration into grading systems, or explaining test takers the relevance and importance of PISA test results (Baumert and Demmrich, 2001; Finn, 2015; Schüttpelz-Brauns et al., 2020). Without applying any strategy to increase test-takers’ effort, researchers found decreased intraindividual effort over the course of taking a test, this time effort of apprentices (technicians, clerks, and lab assistants, Lindner et al., 2018).

Decreasing test-taking effort is a serious problem since (computer-assisted) test-taking performance reflects an unknown amount of the tested ability in this case and threatens validity for the examined sample (Penk and Richter, 2017; Nagy et al., 2018). Furthermore, decreasing test-taking effort in a mock exam might affect achievement related choices (e.g., respond vs. not respond on a computer-assisted task) and the subsequent learning behavior in preparation of the exam. Achievement related choices in computer-assisted tasks regard test-takers’ information processing. Undergraduate students in higher education often have the choice, if they respond on a computer-assisted task in a mock exam, and how much effort they spend on different types of task.

Effort is usually described as a component of achievement motivation (Eccles et al., 1984; Eccles and Wigfield, 1995; Wigfield and Eccles, 2000) or test-taking motivation (Wise and DeMars, 2005; Knekta and Eklöf, 2015; Knekta, 2017). Test-taking motivation is the engagement and effort that a person applies to a goal in order to achieve the best possible result in an achievement test (Wise and DeMars, 2005). Invested effort is conceptualized as relevant predictor on test performance according to the expectancy-value model applied to a test situation (Knekta and Eklöf, 2015, p. 663).

Knekta and Eklöf (2015) investigated adolescents’ test-taking motivation and academic achievement, alongside further motivational components such as test-taking importance, expectancies, anxiety, and interest. A great deal of evidence supports the relevance of these motivational components for test performance (Eklöf and Hopfenbeck, 2018) and academic achievement (e.g., Nagy et al., 2010). For example, self-reported expectancies, test-taking effort, and test-taking importance have been found to determine performance in high-stakes tests (e.g., Knekta, 2017; Eklöf and Hopfenbeck, 2018); as one would expect, high levels of these motivation components predicted higher performance levels. In low-stakes achievement tests, the relations between test-taking effort or test-taking importance (assessed by self-reports) and performance were inconsistently at zero (Sundre and Kitsantas, 2004; Penk and Richter, 2017) to low levels (Knekta, 2017; Eklöf and Hopfenbeck, 2018; Myers and Finney, 2019).

High-stakes achievement tests usually refer to ability assessments for selection purposes (e.g., enrollment in a type of school, internship, study program, or exams to complete a study program, e.g., Knekta, 2017). Low-stakes tests considered in previous research have included tests to practice high stakes-tests (e.g., mock exams), tests to evaluate educational programs (Brunner et al., 2007; Butler and Adams, 2007), tests to develop or update standardized achievement test inventories (e.g., standardization in a representative sample), and cognitive ability tests for research purposes (McHugh et al., 2004; Erle and Topolinski, 2015; Gorges et al., 2016; Goldhammer et al., 2017). Cognitive ability tests for research purposes and tests of subject content knowledge are often used in international large-scale assessments including students in school (Baumert and Demmrich, 2001; Butler and Adams, 2007; Kunina-Habenicht and Goldhammer, 2020) or standardized achievement tests in higher education in the United States (Silm et al., 2020).

**FIGURE 1:**
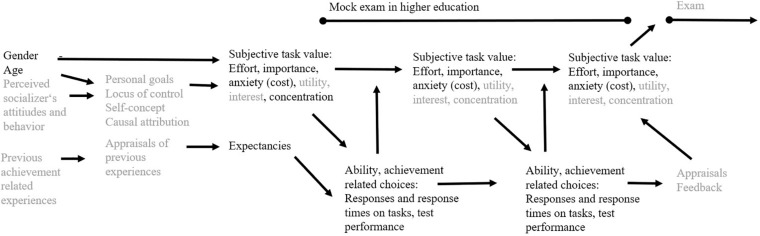
Theoretical model adapted to the mock exam situation in higher education based on previous expectancy-value models (Wigfield and Eccles, 2000; Knekta and Eklöf, 2015). Only constructs written in black font were included in the current study. This study focused on possible decreases of test-taking effort and importance moderated by test battery order with consideration of expectancies, anxiety, and concentration as covariates.

The motivation at the end of a computer-assisted mock exam possibly determines undergraduate students’ exam preparation. The theoretical model in Figure 1 describes both test-taking effort and importance as significant determinants of the upcoming exam. The current study focused on test-taking effort and importance of *education students in higher education in Germany* at three measurement points *during a computer-assisted mock exam moderated by the experimentally varied order of a cognitive ability test and a battery of mock exam tasks*. Hence, the current study aimed to (1) examine whether education students’ test-taking effort and importance decrease over the course of a computer-assisted cognitive ability test and subsequent computer-assisted mock-exam tasks, or vice versa, mock-exam tasks and a subsequent cognitive ability test considering further motivational components as covariates and (2) analyze differences in education students’ information processing and response processes for these two task types depending on their order. The theoretical background of the model in Figure 1 is outlined in the next section.

## Theory and Assessment of Test-Taking Effort and Importance as Motivational Components

One way to disentangle the contributions of ability and motivation is to measure motivation in addition to the ability being tested (e.g., Baumert and Demmrich, 2001; Bensley et al., 2016), based on expectancy-value theory that has its roots in part in motive, expectancy, and incentive as determinants of “aroused motivation to achieve” proposed by Atkinson (1957, p. 362).

### Expectancy-Value Theory

Expectancy-value theory (Atkinson, 1957; Wigfield and Eccles, 2000) summarizes the relations among a number of individual background variables, that are in brief: gender, age, aptitudes, perceived socializer’s beliefs and behavior, subjective appraisal of previous achievement related experiences, affective memories, and self-concept which help explain variance in learners’ achievement related choices. Most of these background variables are out of the current study’s scope except gender and age since Knekta and Eklöf (2015) presented their further developed expectancy-value model applied to a test situation (Knekta and Eklöf, 2015, p. 663). Both theoretical approaches (Wigfield and Eccles, 2000; Knekta and Eklöf, 2015) posit that a person’s achievement related choices are in part explained by gender, age, and the subjective task value which includes a number of motivational components, namely effort, importance, expectancies, and anxiety. Expectancy of success, and subjective task value (i.e., incentive and attainment value, utility, interest).

### Evidence for the Expectancy-Value Theory

A large body of evidence supports the assumptions made in expectancy-value theory. For example, gender consistently explained variance in test-taking effort with females having an advantage over males in a review of literature (DeMars and Bashkov, 2013). The size of the gender gap seems to vary over age groups (DeMars and Bashkov, 2013). Other review results suggested that test-taking effort decreased with increasing years of age (Silm et al., 2020). Another study included undergraduates from 18 to 69 years of age with 56% being 35 years of age or less for investigating their test-taking behavior (Rios and Liu, 2017). The authors discussed the results and limitations of their study as follows: “we evaluated the comparability of proctored groups by gender, ethnicity, language, age, and GPA [grade point average]. We found no significant group differences across all variables except gender and age” (Rios and Liu, 2017, p. 11).

#### Assessment of Test-Takers’ Motivation by a Questionnaire

To assess levels of motivational components, researchers typically use well-established motivation inventories which were developed to measure motivation as a trait (Midgley et al., 1998; Simzar et al., 2015) or state (Vollmeyer and Rheinberg, 2006; Freund and Holling, 2011; Freund et al., 2011). Simzar et al. (2015) and other researchers (Arvey et al., 1990) have found inconsistent relations between trait motivation and test performance (Sundre and Kitsantas, 2004). Indeed, motivation while taking an achievement test is also conceptionally related to a person’s motivational state in that situation. One questionnaire measuring current motivational state is the Questionnaire on Current Motivation (QCM) (e.g., Vollmeyer and Rheinberg, 2006), which several studies (e.g., Penk and Richter, 2017) have used to disentangle the relationship between current motivation, including the dimensions of anxiety, challenge, interest, and probability, and test performance (Freund et al., 2011). Findings from studies using the QCM indicate relations at moderate levels between interest and test scores (Freund et al., 2011). However, one at least partial limitation is that the QCM asks about current motivational state in a general manner. A measurement method closer to the test situation is to ask test takers how they estimate their current motivation before and after taking an achievement test (e.g., Baumert and Demmrich, 2001; Eklöf, 2006).

Eklöf (2006) developed the Test-Taking Motivation Questionnaire (TTMQ), which includes motivational components in line with the expectancy-value theory of achievement motivation (e.g., Wigfield and Eccles, 2000). The relations between these components and test performance were at low to moderate levels, indicating inconsistent findings (Wise and DeMars, 2005; Knekta and Eklöf, 2015; Penk and Schipolowski, 2015; Penk and Richter, 2017; Stenlund et al., 2018). Moreover, Penk and Richter (2017) identified changes in the motivational component of test-taking effort during test taking. Test takers’ self-reported test-taking effort decreased from the beginning to the end of the test in this study (Penk and Richter, 2017) and in other studies (Attali, 2016; Lindner et al., 2018). Test takers may easily recognize that the TTMQ items are intended to capture their motivational state and might thus respond in socially desirable ways. Hence, it is valuable to increase the validity of the TTMQ results by employing less subjective measures (AERA et al., 2014).

#### Time on Task and Response Times as Indicators of Test-Takers’ Motivation

Test-taking effort has been investigated by different measures, for example, response times (Wise and Kong, 2005), time on task (Attali, 2016), or self-reports (Knekta and Eklöf, 2015). A study compared test-taking effort (measured by time on task) and performance in a high-stakes achievement test vs. subsequent low-stakes achievement test with the result that the majority of test takers replicated their high stakes performance in the low-stakes condition with little effort (Attali, 2016).

Some researchers have used response times to test the assumption of low test-taking motivation reflected in low effort (Wise and Kong, 2005; Hartig and Buchholz, 2012; Debeer et al., 2014; Rios et al., 2014), examining persistence levels in terms of response times on puzzle tasks, or response times on anagram tasks (e.g., Gignac and Wong, 2018). Other studies included changes in response times over the course of an achievement test as indicators for test-taking motivation (Hartig and Buchholz, 2012; Goldhammer et al., 2017). Meta-analytic results suggested higher correlations between test-taking response time effort and test performance than self-reported effort assessed mainly by the Student Opinion Scale (Sundre and Moore, 2002) and test performance (Silm et al., 2020). Test-taking effort estimated using response times decreased over the course of test taking in these studies (Hartig and Buchholz, 2012; Debeer et al., 2014).

In summary, changes in self-reported effort over the course of test taking suggest decreased effort, which raises the question of potential strategies for keeping test-taking effort levels. The TTMQ, based on expectancy-value theory, captures current test-taking motivation (state), and is a widely used measure in large-scale assessments including students at school. Researchers examined and proposed strategies with the intention to increase German school students’ test-taking motivation but examined relatively seldom changes in test-taking motivation or strategies to keep the level of test-taking motivation in education students in Germany (Silm et al., 2020). Based on expectancy-value theory and above-mentioned evidence, we focused on two motivational components among test takers: (1) the test-taking effort invested and (2) the subjective test-taking importance of the respective task (value component), while also considering the other components that are expectancies, concentration, and anxiety, as well as gender and age, as described below in the method section. Test-taking effort and importance are probably at higher levels when test takers are working on mock exam tasks than on cognitive ability tasks.

## The Present Research

We aimed to extend the findings on changes in test-taking motivation presented in the previous section (Baumert and Demmrich, 2001; Debeer et al., 2014; Bensley et al., 2016; Knekta, 2017; Penk and Richter, 2017) by employing a computer-assisted experimental design with repeated motivational measures (test-taking effort, test-taking importance) in order to examine changes in these motivational components over experimental variations in task type order, and whether achievement related choices, information processing and response processes are affected by the electronically varied task type order. The purpose was to obtain new insights into possible changes in test-taking effort and test-taking importance across variations in task type order. Test-taking effort and importance were assessed before and after a computer-assisted cognitive ability test and mock exam to obtain insights into intraindividual changes in effort over test taking in a new context (i.e., education students in a computer-assisted environment in higher education) using different measures than in previous studies (e.g., Freund et al., 2011). Moreover, finding different levels of test-taking effort and importance in these conditions would conceptually replicate findings from previous studies on test-taking motivation in other contexts (e.g., Eklöf, 2006; Knekta, 2017). This would extend the validity of test-taking effort and/or importance scores to further test conditions and samples (Knekta and Eklöf, 2015; Penk and Schipolowski, 2015; Knekta, 2017).

Our hypotheses were as follows: (1) Test-taking effort and test-taking importance decrease across three measurement points during the test situation moderated by task type order (first cognitive ability tasks, second mock exam tasks vs. first mock exam tasks, second cognitive ability tasks) and with consideration of the five relevant covariates test expectancies, test anxiety, concentration, gender, and age. (2) Response processes on the ability tests differ depending on the task type order (first cognitive ability tasks, second mock exam tasks vs. first mock exam tasks, second cognitive ability tasks). We included the five relevant covariates in the analyses with regard to Hypothesis 1 since they are considered in the theoretical model (see Figure 1), previous research suggested them as relevant covariates as introduced above (DeMars and Bashkov, 2013; Rios and Liu, 2017; Silm et al., 2020), and covariates are commonly included into experimental designs to reduce variance for increasing statistical power.

To examine our assumptions, we adapted and used measures from previous research (Arvey et al., 1990; Butler and Adams, 2007; Erle and Topolinski, 2015; Knekta and Eklöf, 2015), with the exception of the mock exam tasks. We used items from the Test-Taking Motivation Questionnaire (TTMQ) developed by Eklöf (2006) that has previously been employed in large-scale surveys (e.g., Knekta and Eklöf, 2015), cognitive ability tasks (e.g., Erle and Topolinski, 2015; 10 further tasks for other research purposes, McHugh et al., 2004), and mock exam tasks in the two test order conditions. Similar to other researchers, we analyzed the changes in test-taking motivation over the course of an exam by structural equations, in particular, latent growth curve modeling (e.g., Penk and Richter, 2017). The term “latent” refers to constructs or processes which are not observable. The advantage of measuring factors and their relationships at a latent level is that measurement errors have been separated out.

We additionally analyzed the responses and the response times of the test takers in the cognitive ability tasks and the mock exam tasks with a psychometric diffusion model. The psychometric diffusion model is capable to separate motivational parts from achievement parts of a test taker’s test performance. This provides a more objective basis for the analysis of test takers’ motivation. Psychometric diffusion modeling for these tasks has not yet been undertaken in previously published work. Hence, the current study, with its experimental design, extends previous research on test-taking effort with regard to response processes for different task types.

## Materials and Methods

### Participants

The current study involved *N* = 320 undergraduate education students (77% female, *M*_age_ = 21, *SD*_age_ = 3.13 at T1, seven missing values in gender, one missing value in age) who voluntary attended an electronic mock exam at a German University. The sample size is sufficient for detecting moderate group differences and changes using latent growth curve modeling as simulation studies suggested (Fan, 2003). The electronic mock exam included questions concerning test-taking motivation (presented up to three times), cognitive ability tasks (less personally important tasks), mock exam tasks (personally important tasks), and demographic questions. The mock exam was computerized using the software package PsychoPy (Peirce et al., 2019) and presented on laptops in an e-exam hall. Each undergraduate student used one laptop on a desk with sight protection.

**FIGURE 2:**
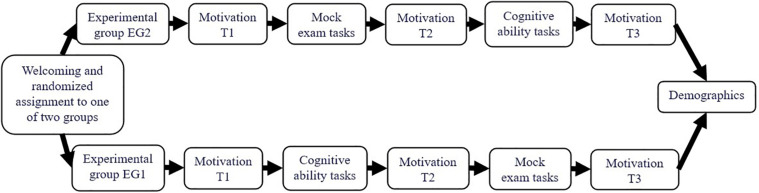
The study design. This study focused on possible changes in test-taking effort and importance. Motivation at Time 1 (T1) included the five factors test-taking effort, test-taking importance, expectancies, anxiety, and concentration according to the theoretical model (see Figure 1). Motivation at Time 2 (T2) and Time 3 (T3) included the factors test-taking effort and test-taking importance.

After welcoming, one of three supervisors read a standardized oral instruction in German aloud for the participants. For example, the instruction involved (in English for the current purposes): “We offer the mock exam for the first time and would like to know how you like it. Therefore, other tasks and a few questions about your motivation are included in addition to the mock exam tasks. Please answer all tasks and questions conscientiously so that the results are meaningful.” The participants further received the information that they may expect 20 mock exam tasks. They could individually decide when to finish a mock exam task and proceed with the next one. There was no time limit. Figure 2 presents the study design. All measures, task descriptions, and tasks were implemented in the programmed experiment using PsychoPy.

The data collection was completely anonymized by assigning the participants electronically generated IDs. There was no deception. All steps of the study followed international ethical standards (AERA et al., 2014). Data from 11 participants were invalid due to technical problems, such as system aborts, and had to be excluded. Thirty-four undergraduate students participated in interventions for other research purposes than presented here, leaving data from *n* = 275 participants remaining for current analyses.

### Measures

The motivational measures employed had already been used in international large-scale surveys (e.g., Arvey et al., 1990; Knekta and Eklöf, 2015). To test the theoretical model introduced (see Figure 1) with the focus on education students’ test-taking effort and importance, we adapted some items to the current study as detailed below. Test-takers’ expectancies, test anxiety, and concentration are included as covariates and assessed once (interest for other research purposes than presented here). Test-taking effort and importance are assessed three times (see Motivation T1, T2, and T3, in Figure 2). Measures only available in English were translated into German using standard cross-translation procedures. All items were presented in German during the mock exam but example items will be translated to English here. Participants indicated their concentration, expectancies, test anxiety, test-taking effort, and importance on rating scales ranging from −1.5 (*strongly disagree*) to 1.5 (*strongly agree*).

We used McDonald’s ω, instead of Cronbach’s α, to estimate the internal consistency of test-taking motivation and each of its dimensions test-taking effort, test-taking importance, expectancies, anxiety, and concentration simultaneously (Dunn et al., 2014). For example, Dunn et al. (2014) argued for McDonald’s ω since it is a point estimate that makes few and realistic assumptions, requires congeneric variables rather than tau-equivalent variables (Zinbarg, 2006; Revelle and Zinbarg, 2008; Hayes et al., 2020). Furthermore, inflation and attenuation of internal consistency estimation are less likely (see Dunn et al., 2014, for further advantages over Cronbach’s α). McDonald’s coefficient can be calculated within the R environment (R Development Core Team, 2009) using the R package *psych* (Revelle, 2019) and interpreted by the same levels as Cronbach’s α (Schweizer, 2011). Note the increasing number of publications about Cronbach’s α vs. McDonald’s ω which consistently suggest McDonald’s ω (Zinbarg, 2006; Revelle and Zinbarg, 2008; Hayes et al., 2020).

#### Motivational Factors

*Test-taking effort* (Knekta and Eklöf, 2015) with regard to the current test situation was measured three times (T1–3) during the mock exam with five items: in the baseline assessment (T1), after the first task battery, and after the second task battery (T3). An example item is “I am doing my best on these tasks.” McDonald’s ω_total_ = 0.95 suggested good internal consistency for the three-factor solution and each factor (T1 ω = 0.87, T2 ω = 0.89, T3 ω = 0.89). Subsequently, items assessing test-taking importance were presented.

*Test-taking importance* (Knekta and Eklöf, 2015) was measured three times (T1–3) with the same three items: in the baseline assessment (T1), after the first task battery and test-taking effort items as well as after the second task battery and effort items (T3). An example item is “The tasks are important to me.” McDonald’s ω_total_ = 0.93 suggested good internal consistency for the three-factor solution as well as the factors test-taking importance at T1 and T3 each except T2 with only acceptable internal consistency (T1 ω = 0.87, T2 ω = 0.60, T3 ω = 0.97).

Moreover, McDonald’s ω_total_ = 0.87 suggested good internal consistency for the motivational five-factors solution incl. expectancies (ω = 0.63), anxiety (ω = 0.74), concentration (ω = 0.69), test-taking effort (ω = 0.87), test-taking importance (ω = 0.87) at T1 and acceptable internal consistency of these factors each. These five motivational variables were included according to the introduced theoretical model (see Figure 1). Subsequent to the test-taking importance items, *expectancies* (Knekta and Eklöf, 2015) were assessed with three items adapted to the current study (T1). An example item is “Compared with other students, I think I am doing well on the tasks.” *Test anxiety* was assessed with three items and presented before the first set of tasks. An example item is “I am so nervous when I take the tasks that I forget things I usually know” (adapted from Knekta and Eklöf, 2015, p. 666). *Concentration* (Arvey et al., 1990) was assessed with four items at the end of the baseline assessment (T1). An example item is “It is hard to keep my mind on this test.” Expectancies, anxiety, and concentration were included as manifest covariates only to consider their effects on the criterion variables test-taking effort and test-taking importance at T3 since the theoretical model and previous findings suggested such relations (Knekta and Eklöf, 2015; Silm et al., 2020).

#### Cognitive Ability Tasks and Mock Exam Tasks

Pioneers of psychology already tested and described cognitive abilities such as perception (James, 1884), reasoning (Piaget, 1928), and visuo-spatial perspective-taking (Flavell et al., 1978). Since perspective-taking is highly important for education students’ social interactions with children, adolescents, and adults (Wolgast et al., 2019), we chose proven cognitive ability tasks as typical tasks for research purposes in psychology. These *cognitive ability tasks* were considered as personally less important low-stakes tasks because they were not part of the lecture or module curriculum and irrelevant for the exam the students had to take in order to finish the course. Sixteen tasks assessed the cognitive ability *visuo-spatial social perspective-taking* that is seeing what another person sees by putting oneself mentally in the target’s spatial position (Kessler and Thomson, 2010; Erle and Topolinski, 2015).

Erle and Topolinski (2015) used the visuo-spatial perspective-taking paradigm developed by Kessler and Thomson (2010). Each of the first 16 tasks involved a photograph (with friendly permission from Thorsten M. Erle for using the photographs in further research). The photograph showed a female or male target person sitting at a round table (arms on the table) from a bird’s-eye perspective. A book and a banana lay on the table close to the person’s left arm and right arm, respectively, or vice versa. The person’s position at the table rotated from photograph to photograph between 120, 160, 200, and 240° from the participant’s point of view. Previous research has found perspective-taking to be difficult at these angles (Janczyk, 2013). Each position was presented with a female target person in eight photographs and a male target person in further eight photographs (16 tasks). Participants indicated whether the book was lying closer to the target person’s left or right arm by pressing “n” (*left*) or “m” (*right*) on the keyboard (“n” is located to the *left* of “m” on German keyboards). There was no time limit. All cognitive ability tasks were presented in German. McDonald’s ω = 0.98 suggested almost perfect internal consistency.

Twenty single-choice *mock exam tasks* were developed to coincide with a lecture for undergraduate education students entitled “Educational Psychology.” McDonald’s ω = 0.61 suggested acceptable internal consistency for these tasks. The mock exam tasks were considered as individually important low-stakes achievement test because the students’ upcoming module exam consisted of tasks of this type with similar content. Hence, the undergraduates had the opportunity to practice this type of task in order to be well prepared for the module exam. An example mock exam task is “Which phenomenon related to a child’s reasoning did Piaget and colleagues investigate with the three-mountain task? (a) object permanence, (b) centering, (c) egocentrism, (d) logical contradictions.” The tasks were presented in German; the example has been translated into English for current purposes.

### Procedure

Participants were randomly assigned to two conditions: EG1 responded first to the cognitive ability tasks and then to the mock exam tasks, while the order was vice versa for EG2 (first mock exam tasks, then cognitive ability tasks). All participants had the opportunity to take the mock exam tasks and subsequently receive automatically generated feedback on how many tasks they solved. The respondents participated voluntarily and gave consent to analyze their data, which was anonymously collected. Taking the tests lasted less than 1 h in total, including initial instruction.

### Statistical Analyses

#### Latent Growth Curve Modeling

We used latent growth curve modeling (R Development Core Team, 2009; Rosseel, 2010) and weighted least squares with mean and variance adjustment estimation (WLSMV) (Rosseel, 2019) to test for within-test-takers’ changes and differences in responses on motivational items depending on condition. We set the significance-level at α = 0.05. The variables included in the modeling were grand mean centered.

**FIGURE 3:**
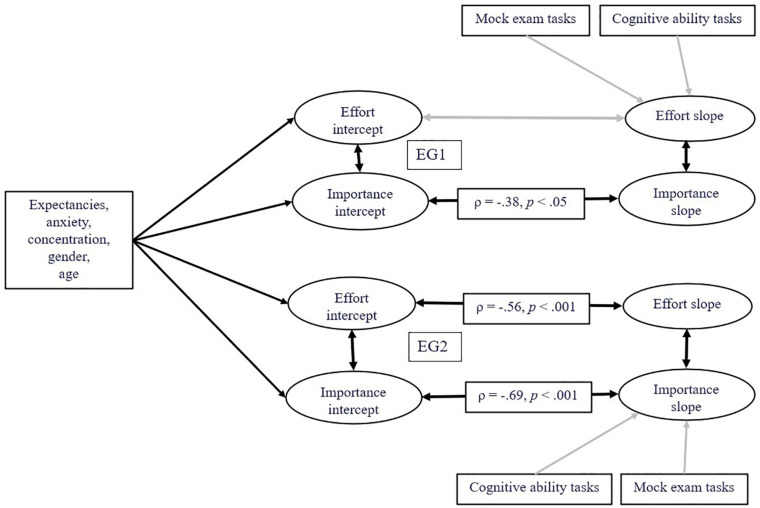
Simplified scheme of the latent growth curve model. ρ = standardized latent correlation coefficient (see Table 4 for standard errors and confidence intervals). Gray arrows represent statistically not significant relations. Latent factors test-taking effort and importance at T1, T2, and T3, indicators and residuals are not depicted in favor of clarity (see Supplementary Figure S2 for the technical model depiction).

First, we conducted a confirmatory factor analysis (CFA) and tested the theoretical six-factor model (test-taking effort and importance at T1, T2, T3) by the data. The unstandardized effort factor-loading of the fourth item (Item “E4,” Knekta and Eklöf, 2015, p. 666, adapted to university: *I spent more effort on this test than I do on other tests we have in university*.) was λ = 0.21 and statistically not significant with *p* = 0.15 in the EG1. Consequently, we excluded Item E4 from further analyses. The final two factor CFA model included the latent factor *test-taking effort* measured by the respective four items and their residuals at T1, T2, and T3, and the latent factor *test-taking importance* measured by the respective three items and their residuals over the three measurement points. Scalar measurement invariance is a prerequisite for latent growth curve modeling. Measurement invariance was tested using the two factor CFA model in a multi-group analysis across groups and time points. This CFA model including constrained factor loadings suggested scalar invariance, Delta Comparative Fit Index (Δ CFI) = 0.004; Delta Root Mean Square Error of Approximation (Δ RMSEA) = 0.003, according to recommended cutoffs (Hu and Bentler, 1999; Svetina and Rutkowski, 2014). The factor structure and intercepts found for EG1’s data were equivalent to the factor structure and intercepts found for the EG2’s data and at T1, T2, and T3. This CFA model is depicted in the Supplementary Figure S1. A simplified version of the second order latent growth curve model for the analysis of individual within-test-takers’ change in test-taking effort and importance is depicted in Figure 3 (see Supplementary Figure S2 for a technical version). The second order latent growth curve model was specified including random intercepts and random slopes by extending the CFA model as follows: At first order latent level, the variance of the factors *test-taking effort* and *importance* each at T1, T2, and T3 has been constrained to the same value for compound symmetry covariance structure (Rosseel, 2019, 2020). At second order latent level, *test-taking effort intercept* has been specified with the three latent factors *test-taking effort* at T1, T2, and T3 with each path fixed to one. The latent factor *test-taking effort slope* has been specified with these three factors and the paths fixed to 0, 1, 2 respectively. The means of expectancies, anxiety, and concentration from the baseline assessment as well as gender and age were included to predict the factor *effort intercept* because these covariates should explain different *effort intercepts* between the education students. The covariates’ category each existed before the study such as gender and age or were assessed before assessing test-taking effort and importance. Gender and age are included as covariates in SEM (Mutz and Pemantle, 2015) since previous findings consistently suggested their relations to test-taking motivation in educational contexts (e.g., DeMars and Bashkov, 2013; Silm et al., 2020). The covariates are included in SEM to consider their anticipated effects on the criterion variables test-taking effort and importance at T3 (see Mutz and Pemantle, 2015, for standards in experimental research).

“A mean structure is automatically assumed, and the observed intercepts are fixed to zero by default, while the latent variable intercepts/means are freely estimated” (Rosseel, 2020, p. 28). The sum scores of the cognitive ability tasks and exam tasks each were included to predict *the test-taking effort slope* (instead of *test-taking effort intercept*) because we experimentally varied the exam tasks’ position. We used the means and sum scores of predictor variables instead of measuring latent factors to keep the number of parameters as low as possible. Random intercepts and slopes for each latent factor were specified with correlations to themselves and to each other. The second-order latent factors *test-taking importance intercept* and *test-taking importance slope* were analogously specified and the same covariates included. Correlations between some test-taking effort indicators were allowed according to modification indices (see Supplementary Figure S2).

The model specification considered EG1’s and EG2’s data separately, so EG1’s data were analyzed without EG2’s data and vice versa. Criterion variables were *latent test-taking effort intercept* and *slope* as well *latent test-taking importance intercept* and *slope*.

#### Latent Diffusion Modeling

To analyze participants’ response processes, we included the responses and response times in a latent trait diffusion model. The diffusion model allows researchers to examine response process components in binary decision tasks (Voss et al., 2013). Binary decision tasks are, for example, the presented cognitive ability tasks where test takers have to choose one of two response options or the questions in the mock exam where the test takers have to decide between the correct and the incorrect response (Molenaar et al., 2015). The diffusion model is based on the assumption that test takers continuously accumulate evidence for the two response options. A momentary preference is formed by weighting the evidence for the two response options against each other. As soon as the momentary preference exceeds a critical level, the test taker responds by selecting the more preferred option.

Diffusion modeling involves defining three process parameters. (1) Information accumulation is a measure of one’s mental simulation of two possible outcomes using the available information (*drift rate*, *v*). (2) The amount of information required for a response is reflected in the decision threshold (*boundary separation parameter*, *a*). (3) The response time includes time for reactions (e.g., moving one’s finger to the keyboard in a computer-based task) and/or other sensory, mental or motor responses aside from the time needed to make a decision (*non-decision time parameter*, *ter*) (Ratcliff and McKoon, 2008; Voss et al., 2013). The drift rate (*v*) provides insights into information uptake latency, with high uptake speed reflecting high performance, and is a manifestation of a test taker’s capability. The lower the drift rate, the more difficult the task is in relation to a given individual latent trait (e.g., ability or attitude, see Voss et al., 2013). Low drift rates are reflected in low response accuracy and long response times. The boundary separation reflects the response caution. It is assumed to be a manifestation of a test taker’s effort or importance, reflecting their carefulness when responding. Low levels of the boundary separation are reflected in low response accuracy and short response times, two typical signs of rapid guessing.

In addition to the parameters in manifest diffusion modeling, the psychometric diffusion modeling under the item response theory allows to estimate the latent person contribution and task contribution to the response process. The person contribution refers to information processing (latent trait θ) and response caution (latent trait ω) of a person as well as investigate relationships between these latent traits (θ, ω) and constructs such as test-taking effort or importance. The task contribution refers to the task difficulty.

Two model types are distinguished, the D-diffusion (Tuerlinckx and De Boeck, 2005) and the Q-diffusion model (Van der Maas et al., 2011). They basically differ in their parameterization. In the D-diffusion model, the effective drift rate is the difference of the latent trait of a test taker and the corresponding intercept. This parameterization allows task probabilities from zero to one. In the Q-diffusion model, the effective drift rate is the quotient of latent ability and the corresponding intercept. The Q-diffusion model requires task solving probabilities of at least 50% for calculating the diffusion parameters. In case of solving probabilities lower than 50%, the D-diffusion model can be used with consideration of its predictions.

We applied diffusion modeling within the R environment (R Development Core Team, 2009) using the R package *diffIRT* (Molenaar et al., 2015). The non-decision time (*ter*) was constrained to control delays resulting from the different laptops we used on the non-response times. The R code can be obtained from the authors.

## Results

**FIGURE 4:**
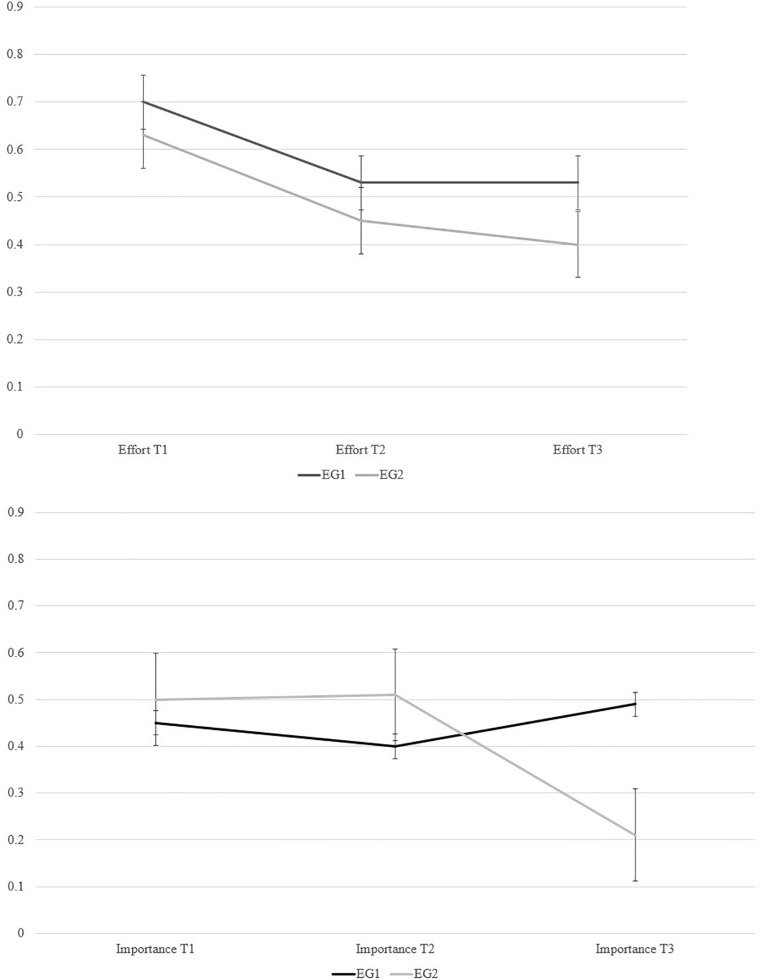
Line diagrams of changes in test-taking effort (above) and test-taking importance (below), means of test takers’ responses (–1.5 = *strongly disagree*, –0.5 = disagree, 0.5 = agree, 1.5 = *strongly agree*, error bars represent standard errors).

Descriptive results are summarized in Tables 1A,B. Product-moment correlations between the variables used are detailed in Table 2. The correlation coefficients suggest zero to low not significant correlations of test-taking effort (T1, T2, T3), test-taking importance (T1, T2, T3), expectancies, anxiety, and concentration with the cognitive ability tasks, and mock exam tasks. Means and standard errors of test takers’ responses on test-taking effort and importance items are depicted in Figure 4. These line diagrams suggested changes in education students’ test-taking effort and importance. For examining these changes at latent level and with consideration of the covariates *expectancies, anxiety*, and *concentration*, we investigated within test-taker effects by structural equations and diffusion modeling.

**TABLE 1A:** Means and standard deviations of the motivational components test-taking effort and test-taking importance in the EG1 (*n* = 125) and EG2 (*n* = 150) at T1, T2, and T3.

	Time 1		Time 2		Time 3	
			
EG1	*M*	*SD*	*M*	*SD*	*M*	*SD*
Effort^*a*^	0.70	0.38	0.53	0.45	0.53	0.40
Importance^*a*^	0.45	0.49	0.40	0.54	0.49	0.44
**EG2**						
Effort^*a*^	0.63	0.37	0.45	0.42	0.16	0.57
Importance^*a*^	0.50	0.47	0.51	0.44	0.21	0.58

**^*a*^Test-taking effort and test-taking importance means of test takers’ responses (−1.5 = strongly disagree, −0.5 = disagree, 0.5 = agree, 1.5 = strongly agree).**

**TABLE 1B:** Mean accuracy and response times on cognitive ability tasks and mock-exam tasks in Experimental Group EG1 (*n* = 125) vs. EG2 (*n* = 150).

	Cognitive ability tasks		Mock exam tasks	
		
EG1	*M*	*SD*	*M*	*SD*
Mean accuracy	94%	17%	57%	88%
Mean rt (s)	33.63	13.62	607.65	163.53
**EG2**				
Mean accuracy	92%	19%	54%	91%
Mean rt (s)	33.97	15.88	692.37	207.04

**TABLE 2:** Correlations among test-taking effort at T1–3, test-taking importance at T1–3, expectancies, test-taking anxiety, concentration, cognitive ability tasks, mock exam tasks, and age in Experimental Group EG1 vs. EG2.

Variable	1	2	3	4	5	6	7	8	9	10	11	12
1 Effort T1		**0.89**	**0.56**	**0.79**	**0.77**	0.48	−**0.64**	0.08	0.13	<0.01	0.04	−0.21
2 Effort T2	**0.73**		**0.68**	**0.80**	**0.86**	**0.59**	−**0.79**	0.02	0.13	−0.02	0.05	−0.22
3 Effort T3	**0.82**	**0.83**		0.52	**0.59**	**0.91**	−**0.64**	−0.27	0.27	−0.16	−0.28	−0.25
4 Importance T1	**0.70**	**0.84**	**0.81**		**0.88**	0.54	−**0.64**	−0.04	0.07	0.04	0.07	−0.05
5 Importance T2	0.54	**0.83**	**0.78**	**0.94**		**0.65**	−**0.71**	0.01	0.13	0.07	−0.02	−0.25
6 Importance T3	**0.65**	**0.79**	**0.88**	**0.92**	**0.93**		−**0.64**	−0.18	0.14	−0.14	−0.22	−0.26
7 Concentration	−**0.68**	−**0.58**	−**0.74**	−**0.58**	−0.48	−**0.61**		−0.11	0.04	−0.15	−0.18	0.19
8 Expectancies	0.18	0.15	0.06	0.17	0.11	0.12	−0.23		−0.41	−0.20	0.28	0.10
9 Anxiety	−0.02	0.12	0.07	0.31	0.32	0.26	0.15	−0.47		−0.09	−0.48	−0.33
10 Cogn. ability	−0.31	−0.35	−0.19	−0.44	−0.37	−0.35	−0.03	−0.39	−0.35		−0.19	−0.26
11 Mock exam	0.09	−0.12	−0.04	−0.10	−0.22	−0.16	−0.30	0.43	−0.47	−0.18		−0.14
12 Age	−0.09	−0.25	−0.13	−0.25	−0.31	−0.17	−0.26	−0.14	−0.08	0.09	0.06	

**EG1 (n = 125) below the diagonal EG2 (n = 150) above the diagonal, p < 0.05 in bold. Cogn. Ability, cognitive ability tasks; T1, Time 1.**

### Within Test-Taker Effects

First, we employed latent growth curve modeling to disclose changes in education students’ test taking *effort* and *importance* over T1, T2, and T3 moderated by condition (EG1: cognitive ability tasks first vs. EG2: mock exam tasks first). The simplified model structure is depicted in Figure 3 (without depiction of residuals and indicators). The goodness of fit between the theoretical model and data was good (Hu and Bentler, 1999; Svetina and Rutkowski, 2014), χ^2^(641) = 731.04, *p* = 0.008, CFI = 0.99, RMSEA = 0.033, 95% CI [0.018, 0.044], SRMR = 0.078 (WLSMV-estimation).

Figure 3 provides results such as standardized latent correlation coefficients and statistical significance levels.

In Table 3, these standardized latent correlation coefficients are presented with standard errors, significance levels and confidence intervals each. Table 3 provides furthermore variances of effort intercept, effort slope, importance intercept, and importance slope. The results suggested significantly decreased test-taking effort (ρ = −0.56, *p* < 0.001) and importance (ρ = −0.69, *p* < 0.001) in EG2 over test-taking time supporting Hypothesis 1.

**TABLE 3A:** Standardized latent regression coefficients and correlation coefficients, standard errors, confidence intervals, from latent growth curve modeling with intercepts and slopes of test-taking effort and test-taking importance in Experimental Group EG1 and EG2.

EG1, *n* = 125	β	*SE*	*p*	CI_lower_	CI_upper_
Effort slope regressed on					
Cognitive ability tasks	0.53	0.38	0.86	−0.51	0.57
Exam tasks	−0.10	0.66	0.88	−0.39	0.19
Importance slope regressed on					
Cognitive ability tasks	0.07	0.08	0.44	−0.10	0.23
Exam tasks	−0.05	0.14	0.72	−0.33	0.23
**EG2**, *n* = 150					
Effort slope regressed on					
Cognitive ability tasks	0.03	0.07	0.68	−0.11	0.17
Exam tasks	−0.05	0.09	0.54	−0.23	0.12
Importance slope regressed on					
Cognitive ability tasks	0.03	0.07	0.69	−0.11	0.16
Exam tasks	−0.03	0.09	0.76	−0.20	0.14

**EG1**	**ρ**	***SE***	***p***	**CI_lower_**	**CI_upper_**

Effort intercept∼slope	0.63	0.57	0.27	−0.48	1.74
Importance intercept∼slope	−0.38	0.18	0.03	−0.73	−0.04
**EG2**					
Effort intercept∼slope	−0.56	0.12	<0.001	−0.79	−0.33
Importance intercept∼slope	−0.69	0.12	<0.001	−0.91	−0.46

**TABLE 3B:** Effort intercept, effort slope, importance intercept, and importance slope: Variances at latent level, and explained variances from latent growth modeling.

EG1	*Va*	*SE*	*p*
Effort intercept variance	0.27	0.14	0.046
Effort slope variance	0.01	0.08	0.95
Importance intercept variance	0.41	0.10	<0.001
Importance slope variance	0.07	0.03	0.04
**EG2**			
Effort intercept variance	0.39	0.10	<0.001
Effort slope variance	0.12	0.03	<0.001
Importance intercept variance	0.54	0.12	<0.001
Importance slope variance	0.12	0.04	0.001

**EG1**	***R*^2^**		

Effort intercept	0.28		
Effort slope	0.30		
Importance intercept	0.22		
Importance slope	0.01		
**EG2**			
Effort intercept	0.19		
Effort slope	0.004		
Importance intercept	0.13		
Importance slope	0.002		

However, the EG1’s test-taking effort did not decrease over time (ρ = 0.63, *p* = 0.27), only their test-taking importance decreased (ρ = −0.38, *p* < 0.05) but less than the EG2’s test-taking importance. No significant relations existed between cognitive ability task performance or mock exam task performance and the factors *test-taking effort slope*, and *test-taking importance slope*.

This model explained 28% of variance in the latent factor effort intercept, 30% in the latent factor effort slope, 83% of variance in the latent factor test-taking effort at T3, 22% of variance in the latent factor importance intercept, 1% in the latent factor importance slope, and 83% in the latent factor importance at T3 in the EG1. In the EG2, this model explained 19% of variance in the latent factor effort intercept, 0.4% in the latent factor effort slope, 86% of variance in the latent factor test-taking effort at T3, 13% of variance in the latent factor importance intercept, 0.2% in the latent factor importance slope, and 84% in the latent factor importance at T3.

The theoretical model adapted to the current study (see Figure 1) implies that achievement related choices can involve decisions in response processes on tasks. We examined the EG1’s vs. EG2’s responses and response times on the cognitive ability tasks and mock exam tasks by diffusion modeling as described next.

### Education Students’ Response Processes for the Tasks

The responses for the cognitive ability tasks as well as response times were included in a Q-diffusion model to analyze the achievement related choices and response processes according to the theoretical model in Figure 1. We investigated the goodness of fit between the theoretical and observed response time distribution with QQ-plots which suggested good fit for both groups (see Supplementary Figure S3 for examples). The average intercept of the boundary separation and the average intercept of the drift rate over the items are summarized in Table 4 for the EG1 and the EG2. A Wald test for the equivalence of the boundary intercepts in the two groups was significant (*X ^2^* = 42.00, *df* = 16, *p* < 0.01). A *post hoc* comparison of the intercepts in the single items revealed that parameters deviated in two of the 16 items on α = 0.05. The corresponding Wald test for the equivalence of the drift intercepts was also significant (*X ^2^* = 37.83, *df* = 16, *p* < 0.01). The drift rates differed in three of the 16 items on α = 0.05. This implies that neither the average response caution, usually considered as a motivational aspect of the response process, nor the average rate of information accumulation, usually considered as an aspect of a test taker’s performance, differed between the groups in most items.

**TABLE 4:** Mean boundary separations (*a*), mean drift rates (*v*), and standard deviations from diffusion modeling including the cognitive ability tasks or mock exam tasks in Experimental Group EG1 vs. EG2.

Tasks	Parameter	EG1		EG2	
			
		*M*	*SD*	*M*	*SD*
Cognitive ability	*a*	0.27	0.03	0.25	0.02
	*v*	0.95	0.14	1.04	0.15
Exam					
	*a*	0.10	0.02	0.09	0.02
	*v*	−0.04	0.10	−0.03	0.09

**a, boundary separation, v, drift rate, ter was constrained to same values. Q-diffusion model has been used for the cognitive ability tasks, D-diffusion model for the exam tasks due to solving probabilities below 50% which the diffIRT-function cannot handle.**

Investigating the D-diffusion model response fit using QQ-plots for the 20 exam tasks suggested acceptable fit between expected and observed distributions in both groups (see Supplementary Figure S3 for examples). A Wald test for the equivalence of the boundary intercepts in the two groups was significant (*X ^2^* = 33.90, *df* = 20, *p* = 0.03). A *post hoc* comparison of the intercepts in the single items revealed that parameters deviated in 5 of the 20 items on α = 0.05. The corresponding Wald test for the equivalence of the drift intercepts was insignificant (*X ^2^* = 22.81, *df* = 20, *p* = 0.29).

We included the latent information processing ω (speed) and response caution θ (trait) from diffusion modeling as criterion variables in general linear modeling to examine whether they related to the condition (i.e., cognitive ability tasks or mock exam tasks first), baseline variables (i.e., expectancies, anxiety, concentration, test-taking effort, test-taking importance) gender, and age. For the cognitive ability tasks, the latent information processing ω and response caution θ did not relate to the baseline variables expectancies, anxiety, concentration, test-taking effort, test-taking importance, gender, and age with all eight regression coefficients close to zero (*B*_ω_ = −0.04 to 0.01, *B*_θ_ = −0.01 to 0.02) except condition that related to response caution (*B*_θ_ = 0.15, *p* = 0.05) with higher response caution in the EG1 than EG2. Including latent information processing ω and response caution θ with the baseline variables into the model to predict test-taking effort and importance at T3 yielded significant relations between concentration (*B*_ei_ = −0.32, *p* < 0.001), test-taking effort (*B*_ei_ = 0.48, *p* < 0.001), and importance (*B*_ei_ = 0.74, *p* < 0.001) at T1 as well as condition (*B*_ei_ = 2.80, *p* < 0.001) to test-taking effort and importance at T3 with EG1 having an advantage. While concentration levels were higher in the EG2 than EG1, test-taking effort and importance levels were higher in the EG1 than EG2. This model explained 36% of variance in test-taking effort and importance, *F* (10, 252) = 15.82, *p* < 0.001.

For the mock exam tasks, the latent information processing ω and response caution θ did not relate to the baseline variables expectancies, anxiety, concentration, test-taking effort, test-taking importance, gender, and age with all eight regression coefficients close to zero (*B*_ω_ = −0.02 to 0.01, *B*_θ_ = −0.05 to 0.01). Including latent information processing ω and response caution θ with the baseline variables into the model to predict test-taking effort and importance at T3 yielded significant relations between concentration (*B*_ei_ = −0.33, *p* = 0.002), test-taking effort (*B*_ei_ = 0.47, *p* < 0.001), and importance (*B*_ei_ = 0.74, *p* < 0.001) at T1 as well as condition (*B*_ei_ = 2.73, *p* < 0.001) to test-taking effort and importance at T3 with EG1 having an advantage. This model explained 36% of variance in test-taking effort and importance, *F* (10, 252) = 15.63, *p* < 0.001. While concentration levels were higher in the EG2 than EG1, test-taking effort and importance levels were higher in the EG1 than EG2. This model explained 36% of variance in test-taking effort and importance at T3, *F* (10, 252) = 15.63, *p* < 0.001.

## Discussion and Conclusion

Based on expectancy-value theory (Wigfield and Eccles, 2000; Knekta and Eklöf, 2015), the current study sought to examine (1) whether test-taking effort and test-taking importance decrease across three measurement points during the computer-assisted test situation moderated by test-battery order and with consideration of the five covariates test expectancies, test anxiety, concentration, gender, and age and (2) whether response processes on the ability tests and mock exam differ depending on the task type order (EG1: first cognitive ability tasks, second mock exam tasks vs. EG2: first mock exam tasks, second cognitive ability tasks). The response processes refer to the achievement related choices depicted in Figure 1 and regard information processing. Thus, both hypotheses focus on the education students’ test-taking behavior in a computer-assisted environment. Self-reported test-taking effort and importance provide subjective information about test-taking behavior in the computer-assisted environment. Information processing and response processes in tasks involve responses and response times that provide rather objective information about test-taking behavior than self-reported test-taking effort and importance.

The results from latent growth curve modeling suggested that test-taking effort and importance in EG2 significantly decreased among the education students over different task type orders (first mock exam tasks, then cognitive ability tasks) in the computer-assisted environment. Test-taking effort significantly decreased almost linearly from T1 over the mock exam tasks and cognitive ability tasks to T3 in EG2. These declines are in accordance with Hypothesis 1. Test-taking importance significantly decreased in EG1 (moderate effect) and EG2 (strong effect) (Cohen, 1988). Previous findings suggested that test-taking effort and importance changed even over the course of low-stakes testing (e.g., Penk and Richter, 2017). The decline in test-taking effort and test-taking importance in EG2 conceptually replicates similar findings from previous studies including students in school and different tasks (e.g., Knekta, 2017; Penk and Richter, 2017).

However, test-taking effort in the current study did not decrease when cognitive ability tasks were presented first (EG1) in the computer-assisted environment. The results support Hypothesis 1 in part since test-taking effort and importance significantly decreased in EG2 with higher levels when working on mock exam tasks than on the subsequent cognitive ability tasks. Test-taking importance also decreased in EG1 but without an advantage for the mock exam tasks. EG1’s not decreased test-taking effort contradicts Hypothesis 1. Note that test-taking effort and importance were assessed by computer-assisted self-report measures. Diffusion modeling allows more objective insights into response processes in the sense of achievement related choices and information processing while working on tasks.

For the mock exam tasks, boundary intercepts suggesting response caution θ (latent trait and motivational aspect) were similar in both groups. This result implies similar motivational levels in both groups while working on the mock exam tasks. For the cognitive ability tasks, boundary intercepts suggesting response caution θ significantly differed between the groups. Response caution θ related to the condition with higher response caution in EG1 than EG2. Thus, EG1’s motivational levels were higher than EG2’s motivational levels from a more objective point of view than self-reports. This difference is in accordance with Hypothesis 2 and with the result that test-taking effort did not decrease in EG1.

The new finding here is that the education students invested similar test-taking effort in the cognitive ability tasks as in the subsequent mock exam tasks (EG1). In EG1, test-taking effort did not decrease over the task types. Latent diffusion modeling (Van der Maas et al., 2011; Voss et al., 2013) suggested similar response processes on mock exam tasks but differences in the response processes (boundary intercepts) on cognitive ability tasks suggesting higher objective motivational levels in EG1 than EG2.

Latent diffusion modeling has not been undertaken in previous research on test-taking motivation in low-stakes tests. The high accuracy on the computer-assisted cognitive ability tasks might be one explanation approach for the similar response processes on the computer-assisted mock exam tasks between conditions. Another explanation might be that the mock exam tasks predominantly required recalling subject content knowledge of educational psychology and rarely knowledge transfer to educational practice. Changes in education students’ test-taking effort might affect tasks in other computer-assisted environments than presented here which require knowledge transfer to contexts in practice because such transfer is known as cognitively difficult. Alternatively, the testing time of about 30 min was too short for a test-taking effort decline related to a cognitive performance decline.

We concluded from the results, the education students were able to keep their self-reported test-taking effort levels during computer-assisted cognitive tasks and subsequent computer-assisted mock exam tasks. Diffusion modeling suggested objectively measured higher motivational levels during the cognitive tasks when they were presented first (EG1) than when mock exam tasks were presented first (EG2). The education students were not able to keep their test-taking effort during the computer-assisted cognitive ability tasks following the computer-assisted mock exam tasks.

Weak or non-existing relations between the motivational components (assessed by self-reports) and performance in low-stakes achievement tests are already known from other studies that presented relations inconsistently at zero (Sundre and Kitsantas, 2004; Penk and Richter, 2017) to low levels (Knekta, 2017; Eklöf and Hopfenbeck, 2018; Myers and Finney, 2019). The weak or non-existing relations between the motivational components on the cognitive ability tasks and mock exam tasks might have resulted from low to moderate task difficulties which not require to be motivated for performing equally in both conditions.

### Limitations and Implications for Future Research

Participants in the current study were education students and a self-selected sample tested in an e-exam hall (one person per laptop); however, each participant was randomly assigned to one of two experimental conditions. Gender was not equally distributed in the study. The computer-assisted cognitive ability tasks were tasks for research purposes rather than widely used standardized inventories. The computer-assisted mock exam was developed based on the participating students’ educational psychology curriculum, including somewhat broad fundamentals of cognitive psychology, developmental psychology, and social psychology. Consequently, the relatively low internal consistency measured by McDonald’s ω might reflect the curriculum’s broad content. Despite these limitations, however, the present study contributes to the understanding of motivation-performance patterns during computer-assisted test taking in higher education. This finding might be also relevant for motivation, information processing and responses on online exam tasks and online self-assessment tasks. The described differences between the conditions might be considered in the development of new computer-assisted (online) task batteries for exams or self-assessments, especially their order.

Future research might include a within-subject design and computer-assisted (online) tests accompanied by measures assessing test-taking effort and importance before and after the respective test (e.g., effort with regard to Test 1 assessed before Test 1 and subsequently to Test 1, then effort with regard to Test 2 assessed before Test 2 and subsequently to Test 2). It is important for further studies to examine test-taking effort and importance during different ability tasks, because responses on tasks other than those presented here may differently stimulate information processing and differently relate to test-taking effort and importance. Hence, future research should examine the relations between test-taking effort, test-taking importance, and responses on different computer-assisted (online) ability tasks to increase the validity of the presented results (AERA et al., 2014). The current study increased its validity by using test-taking-effort and importance measures, because the changes found support the hypothesis that states should be measured rather than traits (Eklöf, 2006; AERA et al., 2014).

The main contribution of the empirical work presented here is that test-taking effort and importance were assessed three times over an experimentally varied task battery order considering information processing and response processes in a computer-assisted environment in higher education. Roughly 20 years ago, Baumert and Demmrich (2001) presented insignificant findings on strategies to increase students’ test-taking motivation in PISA. However, from this study’s perspective, the more important question is how to keep test-taking effort and importance relatively stable and avoid declines, rather than discussing how to increase test-taking motivation, as has been the case in previous research (e.g., Baumert and Demmrich, 2001).

## Data Availability Statement

The datasets presented in this study can be found in online repositories. The names of the repository/repositories and accession number(s) can be found below: https://osf.io/nmxq7/?view_only=f150703b9a664d648c772055aa3335b3.

## Ethics Statement

Ethical review and approval was not required for the study on human participants in accordance with the local legislation and institutional requirements. The participants provided their written informed consent to participate in this study.

## Author Contributions

AW, NS, and JR contributed in collaboration to the design, conception, and data collection of the study. NS wrote a test documentation including sample and measure descriptions as well as descriptive results presented in the current report. AW analyzed the data by structural equations and latent diffusion models, and wrote the first draft of the manuscript. JR reviewed the manuscript contributing significantly to improve it, in particular by also calculating diffusion models and writing parts of the section about diffusion modeling. All authors contributed to the manuscript revision, reread and approved the current version.

## Conflict of Interest

The authors declare that the research was conducted in the absence of any commercial or financial relationships that could be construed as a potential conflict of interest.

## Supplementary Material

Supplementary Figure 1 CFA-model including test-taking effort and test-taking importance at T1, T2, and T3.

Supplementary Figure 2 Latent growth curve model.

Supplementary Figure 3 QQ-plots to investigate goodness of fit in diffusion modeling including **(A)** cognitive ability tasks and **(B)** mock exam tasks.

Supplementary Table 1 Standardized regression coefficients, standard errors, confidence intervals, and correlations from latent growth curve modeling with intercepts and slopes of test-taking effort and test-taking importance, and covariates in both groups.
