# Supplementary material for: Test-Taking Motivation in Education Students: Task Battery Order Affected Within-Test-Taker Effort and Importance
Source: Front Psychol. 2020 Nov 25;11:559683. doi: 10.3389/fpsyg.2020.559683 (PMC7724091; doi:10.3389/fpsyg.2020.559683)
Supplement: Supplementary Figure 1 — CFA-model including test-taking effort and test-taking importance at T1, T2, and T3. [file Data_Sheet_1.doc]

Supplementary Material

## Supplementary Table

**Table S1**

Standardized regression coefficients, standard errors, confidence intervals, and correlations from latent growth curve modeling with intercepts and slopes of test-taking effort and test-taking importance, and covariates in Experimental Group EG1 vs. EG2.

| **EG1**, *n* = 125 |  | **β** | ***SE*** | ***p*** | **CI_lower_** | **CI_upper_** |
| --- | --- | --- | --- | --- | --- | --- |
| Effort intercept regressed on |  |  |  |  |  |  |
| Expectancies |  | .29 | .11 | .01 | .07 | .52 |
| Anxiety |  | .27 | .13 | .04 | .01 | .52 |
| Concentration |  | -.45 | .12 | < .001 | -.68 | -.22 |
| Gender |  | -.13 | .10 | .19 | -.33 | .06 |
| Age |  | -.06 | .11 | .59 | -.28 | .16 |
| Effort slope regressed on |  |  |  |  |  |  |
| Cognitive ability tasks |  | .53 | .38 | .86 | -.51 | .57 |
| Exam tasks |  | -.10 | .66 | .88 | -.39 | .19 |
| Importance intercept regressed on |  |  |  |  |  |  |
| Expectancies |  | .26 | .08 | .002 | .10 | .42 |
| Anxiety |  | .39 | .08 | < .001 | .23 | .55 |
| Concentration |  | -.24 | .08 | .002 | -.39 | -.09 |
| Gender |  | .02 | .10 | .86 | -.19 | .22 |
| Age |  | -.04 | .09 | .68 | -.22 | .15 |
| Importance slope regressed on |  |  |  |  |  |  |
| Cognitive ability tasks |  | .07 | .08 | .44 | -.10 | .23 |
| Exam tasks |  | -.05 | .14 | .72 | -.33 | .23 |
| **EG2**, *n*= 150 |  |  |  |  |  |  |
| Effort intercept regressed on |  |  |  |  |  |  |
| Expectancies |  | .16 | .07 | .03 | .02 | .30 |
| Anxiety |  | .20 | .09 | .02 | .03 | .37 |
| Concentration |  | -.34 | .08 | < .001 | -.50 | -.18 |
| Gender |  | .15 | .07 | .04 | .01 | .29 |
| Age |  | .04 | .04 | .43 | -.05 | .12 |
| Effort slope regressed on |  |  |  |  |  |  |
| Cognitive ability tasks |  | .03 | .07 | .68 | -.11 | .17 |
| Exam tasks |  | -.05 | .09 | .54 | -.23 | .12 |
| Importance intercept regressed on |  |  |  |  |  |  |
| Expectancies |  | .12 | .06 | .07 | -.01 | .24 |
| Anxiety |  | .15 | .08 | .07 | -.01 | .31 |
| Concentration |  | -.25 | .07 | < .001 | -.38 | -.12 |
| Gender |  | .19 | .08 | .01 | .04 | .34 |
| Age |  | .07 | .05 | .14 | -.02 | .17 |
| Importance slope regressed on |  |  |  |  |  |  |
| Cognitive ability tasks |  | .03 | .07 | .69 | -.11 | .16 |
| Exam tasks |  | -.03 | .09 | .76 | -.20 | .14 |

##
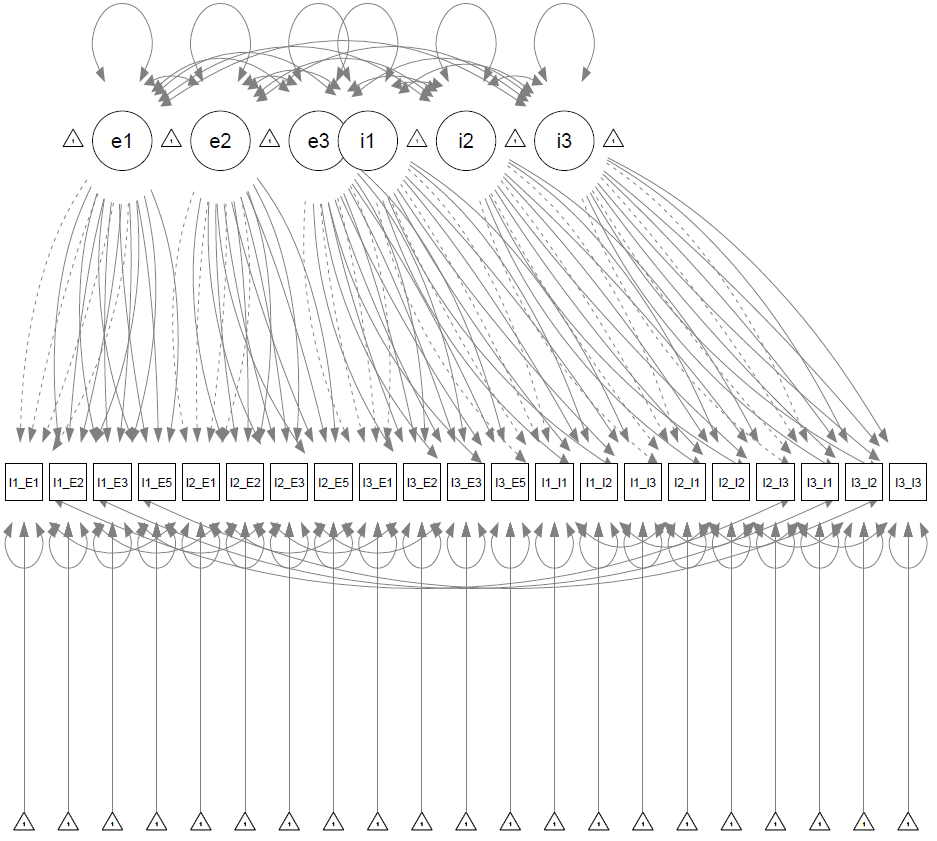
Supplementary Figures and Results from Measurement Invariance Tests

## Supplementary Figure S1 CFA-model including the two latent factors test-taking effort and importance at Time 1, Time 2, and Time 3 (e1–e3, i1–i3). Measurement invariance was tested using this two-factor CFA model in a multi-group analysis. This CFA model including constrained factor loadings suggested scalar invariance, Delta Comparative Fit Index (Δ CFI) = .004; Delta Root Mean Square Error of Approximation (Δ RMSEA) = .003, according to recommended cutoffs (Hu & Bentler, 1999; Svetina & Rutkowski, 2014). The factor structure and intercepts found for EG1’s data were equivalent to the factor structure and intercepts found for EG2’s data at Time 1, Time 2, and Time 3.


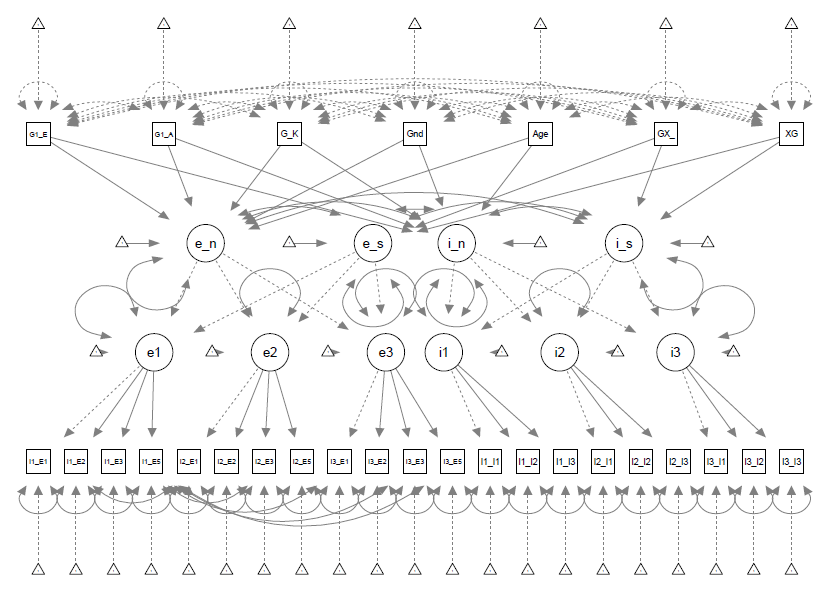


**Supplementary Figure S2** Latent growth curve model including the specified latent factors e1–e3 = effort at T1–T3 respectively, i1–i3 = importance at T1–T3 respectively (and their indicators below), e_n = effort intercept, e_s = effort slope, i_n = importance intercept, i_s = importance slope, and covariates at T1: G1_E = expectancies, G1_A = anxiety, G_K = concentration, Gnd = gender, Age = age, GX_ = perspective tasks, XG = mock exam tasks. Solid lines represent freely estimated relations. Dashed lines represent constraints.

| 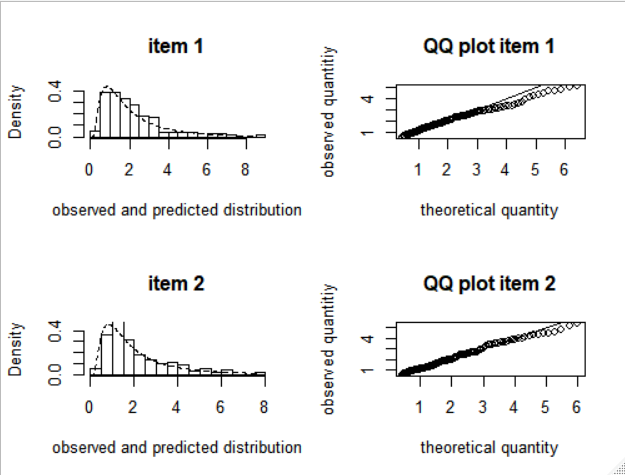 | 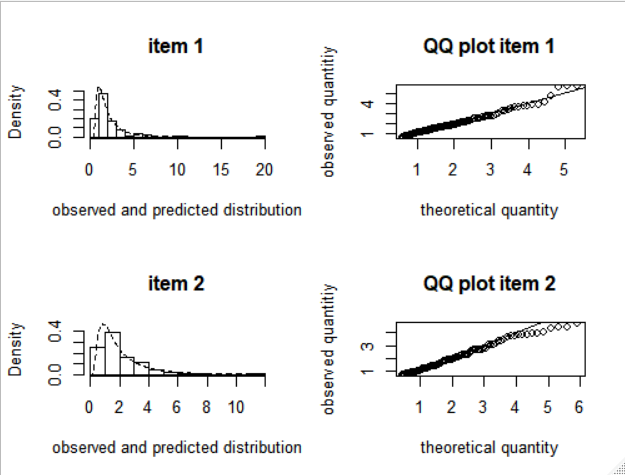 |
| --- | --- |

## Supplementary Figure S3a Theoretical and observed response time distribution of the likelihood ratio statistic (Molenaar et al., 2015) of photo tasks, two examples for EG1 at left and EG2 at right.

| 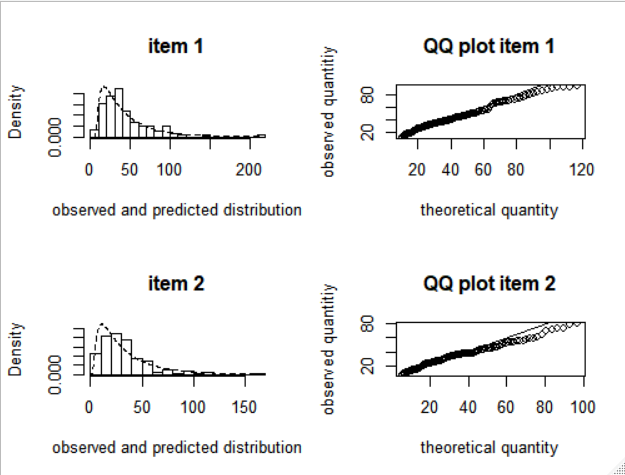 | 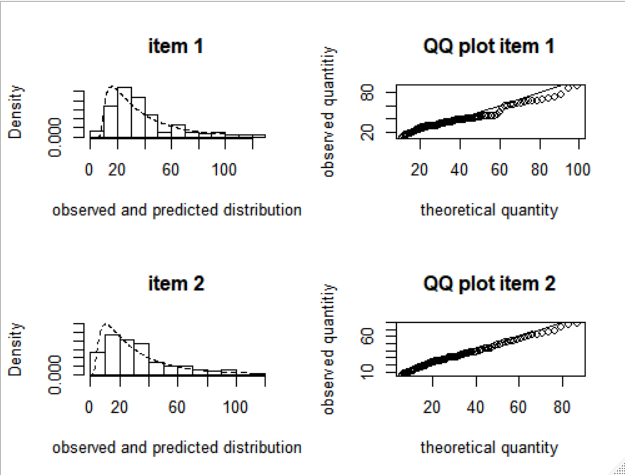 |
| --- | --- |

## Supplementary Figure S3b Theoretical and observed response time distribution of the likelihood ratio statistic (Molenaar et al., 2015) of mock exam tasks, two examples for EG1 at left and EG2 at right.
